# Napabucasin Drug‐Drug Interaction Potential, Safety, Tolerability, and Pharmacokinetics Following Oral Dosing in Healthy Adult Volunteers

**DOI:** 10.1002/cpdd.961

**Published:** 2021-06-09

**Authors:** Xiaoshu Dai, Michael D. Karol, Matthew Hitron, Marjie L. Hard, Matthew T. Goulet, Colleen F. McLaughlin, Scott J. Brantley

**Affiliations:** ^1^ Clinical Pharmacology and Drug Metabolism Sumitomo Dainippon Pharma Oncology, Inc. Cambridge Massachusetts USA; ^2^ Clinical Development Sumitomo Dainippon Pharma Oncology, Inc. Cambridge Massachusetts USA; ^3^ Clinical Pharmacology Nuventra, Inc. Durham North Carolina USA; ^4^ Current address: Praxis Precision Medicines Cambridge Massachusetts USA; ^5^ Current address: Program Management Department KSQ Therapeutics Cambridge Massachusetts USA; ^6^ Pharmacokinetics Nuventra, Inc. Durham North Carolina USA; ^7^ Clinical NCA & PK/PD Nuventra, Inc. Durham North Carolina USA

**Keywords:** breast cancer resistance protein transporter, cytochrome P450, drug‐drug interactions, napabucasin, phase 1 trial

## Abstract

Napabucasin is an orally administered reactive oxygen species generator that is bioactivated by the intracellular antioxidant nicotinamide adenine dinucleotide phosphate:quinone oxidoreductase 1. Napabucasin induces cell death in cancer cells, including cancer stem cells. This phase 1 study (NCT03411122) evaluated napabucasin drug‐drug interaction potential for 7 cytochrome P450 (CYP) enzymes and the breast cancer resistance protein transporter/organic anion transporter 3. Healthy volunteers who tolerated napabucasin during period 1 received probe drugs during period 2, and in period 3 received napabucasin (240 mg twice daily; days 1‐11) plus a phenotyping cocktail containing omeprazole (CYP2C19), caffeine (CYP1A2), flurbiprofen (CYP2C9), bupropion (CYP2B6), dextromethorphan (CYP2D6), midazolam (CYP3A) (all oral; day 6), intravenous midazolam (day 7), repaglinide (CYP2C8; day 8), and rosuvastatin (breast cancer resistance protein/organic anion transporter 3; day 9). Drug‐drug interaction potential was evaluated in 17 of 30 enrolled volunteers. Napabucasin coadministration increased the area under the plasma concentration–time curve from time 0 extrapolated to infinity (geometric mean ratio [90% confidence interval]) of caffeine (124% [109.0%‐141.4%]), intravenous midazolam (118% [94.4%‐147.3%]), repaglinide (127% [104.7%‐153.3%]), and rosuvastatin (213% [42.5%‐1068.3%]) and decreased the area under the plasma concentration–time curve from time 0 extrapolated to infinity of dextromethorphan (71% [47.1%‐108.3%]), bupropion (79% [64.6%‐97.0%]), and hydroxybupropion (45% [15.7%‐129.6%]). No serious adverse events/deaths were reported. Generally, napabucasin is not expected to induce/inhibit drug clearance to a clinically meaningful degree.

Napabucasin (Figure 1) is an orally administered reactive oxygen species (ROS) generator that is bioactivated by the intracellular antioxidant nicotinamide adenine dinucleotide phosphate:quinone oxidoreductase 1 (NQO1).^1^ Cancer cells, including cancer stem cells, often express high levels of NQO1 compared with healthy cells.^2^ Napabucasin exerts its antitumor activity by increasing levels of ROS beyond a cytotoxic threshold, causing cancer cell death.^1^, ^3^ In vitro assays have shown that NQO1‐expressing cancer cells are more sensitive to napabucasin.^1^, ^3^ Several clinical trials have investigated napabucasin as a single agent or in combination with several anticancer treatments (data reported in congress abstracts^4^, ^5^, ^6^, ^7^, ^8^, ^9^, ^10^, ^11^, ^12^, ^13^, ^14^, ^15^ or articles^16^, ^17^, ^18^, ^19^). A phase 3 study of napabucasin in combination with 5‐fluorouracil, leucovorin, and irinotecan vs 5‐fluorouracil, leucovorin, and irinotecan alone in patients with previously treated metastatic colorectal cancer (NCT02753127) is ongoing.^20^

**Figure 1:**
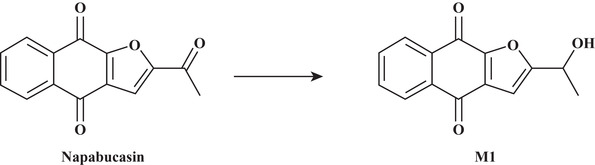
The structure of napabucasin and its major metabolite, dihydro‐napabucasin (M1).

Preclinical studies in bile duct cannulated rats have shown that napabucasin is highly membrane permeable and rapidly absorbed, with relatively high bioavailability (75.5%) following a single oral radiolabeled dose. Metabolism of napabucasin is qualitatively similar between species based on in vitro metabolic studies in cryopreserved hepatocytes from rats, dogs, rabbits, and humans (Sumitomo Dainippon Pharma Oncology, Inc., data on file). The major human metabolite of napabucasin is dihydro‐napabucasin (M1), which has reduced activity compared with napabucasin (Figure 1), reflected by reduced binding to NQO1 with a similar reduction in catalytic efficiency in a cell‐free system. Further, in a human lung cancer cell line (A549 cells), treatment with the M1 metabolite failed to produce ROS (a >10‐fold reduction compared with napabucasin) and diminished reduction of cell viability compared with treatment with napabucasin (Sumitomo Dainippon Pharma Oncology, Inc., data on file). Excretion of radioactivity following administration of a single radiolabeled napabucasin dose is rapid and primarily via the renal and fecal routes in rats, and the fecal route in dogs, with >90% of the dose eliminated within 48 and 24 hours after dosing, respectively (Sumitomo Dainippon Pharma Oncology, Inc., data on file). Absorption, metabolism, excretion, and pharmacokinetics (PK) of a single oral dose of napabucasin in humans was investigated in a phase 1 study (NCT03525405; manuscript in progress).

Patients with cancer often require multiple medications to manage disease symptoms, cancer drug side effects, and possible comorbid conditions, and are therefore susceptible to drug‐drug interactions (DDIs). DDIs increase the complexity of therapeutic management and can induce the development of adverse events (AEs) and/or impact clinical efficacy.^21^, ^22^, ^23^, ^24^, ^25^ In vitro data from preclinical studies in human liver microsomes indicate that napabucasin is an inhibitor of cytochrome P450 (CYP) 1A2, 2B6, 2C8, 2C9, 2C19, 2D6, and 3A4 isozymes, with half maximal inhibitory concentration (IC_50_) values of 0.207, 1.80, 1.02, 5.44, 1.70, 2.35, 1.56 (CYP3A4 probed with testosterone), and 1.37 μmol/L (CYP3A4 probed with midazolam), respectively (Sumitomo Dainippon Pharma Oncology, Inc., data on file). Additionally, in vitro data in porcine (LLC‐PK1), human (HEK293), or *Drosophila melanogaster* (S2) transporter‐expressing cell lines show that napabucasin is an inhibitor of the breast cancer resistance protein (BCRP) transporter (in LLC‐PK1 cells), organic cation transporter 2 (in HEK293 cells), organic anion transporters (OAT) 1 and 3 (in S2 cells), and multidrug and toxin extrusion protein 1 (in HEK293 cells) with IC_50_ values of 1.24, 9.56, 1.74, 0.724, and 11.7 μmol/L, respectively (Sumitomo Dainippon Pharma Oncology, Inc., data on file). In vivo maximum observed plasma concentration (C_max_) of napabucasin in healthy male volunteers after a single oral dose of napabucasin 240 mg is 1.78 μM (geometric mean; manuscript in progress), which is in the range of the IC_50_ values observed in vitro. The primary objective of this open‐label, phase 1 study was to quantitatively evaluate the DDI potential of napabucasin and its major metabolite (M1) (Figure 1) with respect to 7 major CYP enzymes and the BCRP transporter in healthy volunteers. DDIs were assessed using a phenotyping cocktail approach for the probe drugs, which enabled assessment of napabucasin DDI via several different pathways in a single study. Napabucasin was dosed orally to steady state, which is clinically relevant and which facilitated the examination of metabolite effects.

## Methods

### Study Design

The protocol was approved by the institutional review board at the study site (Avail Clinical Research, Deland, Florida) and all healthy volunteers provided written informed consent before participation.

This phase 1, single‐center, open‐label, single‐sequence, 3‐period, PK DDI study (NCT03411122) evaluated the effect of napabucasin on 8 probe drugs (7 CYP probe substrates and 1 BCRP transporter substrate) in healthy volunteers. The probe drugs were caffeine (CYP1A2 substrate), bupropion (CYP2B6 substrate), repaglinide (CYP2C8 substrate), flurbiprofen (CYP2C9 substrate), omeprazole (CYP2C19 substrate), dextromethorphan (CYP2D6 substrate), midazolam (CYP3A substrate), and rosuvastatin (BCRP/OAT3 substrate) (Table 1).

**Table 1:** DDI Probe Drugs (Related CYP or Transporter)

Drug	Dose (mg)	Administration Route
Caffeine (CYP1A2)^a^	100	Oral
Bupropion (CYP2B6)^a^	150	Oral
Repaglinide (CYP2C8)	0.25	Oral
Flurbiprofen (CYP2C9)^a^	50	Oral
Omeprazole (CYP2C19)^a^	20	Oral
Dextromethorphan (CYP2D6)^a^	30	Oral
Midazolam (CYP3A)^a^	2	Oral/IV
Rosuvastatin (BCRP/OAT3)	10	Oral

BCRP, breast cancer resistance protein; CYP, cytochrome P450; DDI, drug‐drug interaction; IV, intravenous; OAT3, organic anion transporter 3.

^a^ Part of the phenotyping cocktail (oral doses only).

### Healthy Volunteers

Healthy male or female adult volunteers aged 18 to 45 years with a body mass index of 18 to 34 kg/m^2^ were eligible for inclusion. Additional eligibility requirements included normal (or abnormal and clinically insignificant according to the investigator) laboratory values at screening and no significant abnormalities at the baseline physical examination. Healthy volunteers also agreed to abstain from taking any dietary supplements, herbal products, or nonprescription drugs (except as authorized by the investigator and medical monitor) and any foods with a known DDI impact (grapefruit, grapefruit juice, Seville oranges, and grapefruit‐ or Seville orange–containing products). Healthy volunteers also abstained from tobacco‐ and nicotine‐containing products, caffeine‐ or chocolate‐containing products, and alcohol‐containing beverages for up to 2 months before study admission through follow‐up. Healthy volunteers were excluded if they had a history of illicit drug abuse or positive findings on urine drug screen; were positive for human immunodeficiency virus, hepatitis B, and/or hepatitis C at screening; were poor metabolizers (ie, 2 inactive allele variants) for CYP2C19, CYP2C9, and/or CYP2D6; or had a hypersensitivity or allergy to napabucasin, any of the probe drugs or their ingredients, or other clinically significant allergy.

This study was conducted in compliance with the Declaration of Helsinki; the ethical principles of Good Clinical Practice, according to International Conference on Harmonization, Harmonized Tripartite Guideline; and applicable national and local regulatory requirements.

### Study Drug Administration

The selection of the probe drugs and doses used in the phenotyping cocktail was based on previous DDI studies conducted in healthy adult volunteers.^26^, ^27^, ^28^, ^29^, ^30^, ^31^, ^32^, ^33^, ^34^, ^35^, ^36^, ^37^ Napabucasin 240 or 480 mg was administered orally twice daily (every 12 hours); healthy volunteers received the morning dose following an overnight fast, remained fasted for 4 hours after dosing, and fasted 1 to 2 hours before administration of the evening dose. Potential DDIs were assessed on the basis of the napabucasin 240‐mg dose only; DDIs were not assessed for the 480‐mg dose because healthy volunteers did not receive the 480‐mg twice‐daily regimen in conjunction with the probe drugs (due to a protocol amendment on October 12, 2017, that adjusted the dose to 240 mg).

Single doses of the phenotyping cocktail and then later repaglinide 0.25 mg and rosuvastatin 10 mg were administered orally with 240 mL of water following an overnight fast; healthy volunteers remained on an empty stomach for 4 hours after dosing. Intravenous (IV) midazolam 2 mg was administered over 1 minute and immediately followed by a 5‐mL normal saline IV bolus to flush the indwelling catheter. Water was permitted ad libitum for all doses except 1 hour before and after drug administration on fasting days; healthy volunteers remained at rest for 2 hours after dose administration (except in the event of AEs and for study procedures [eg, IV midazolam administration]).

### Study Procedures

**Figure 2:**
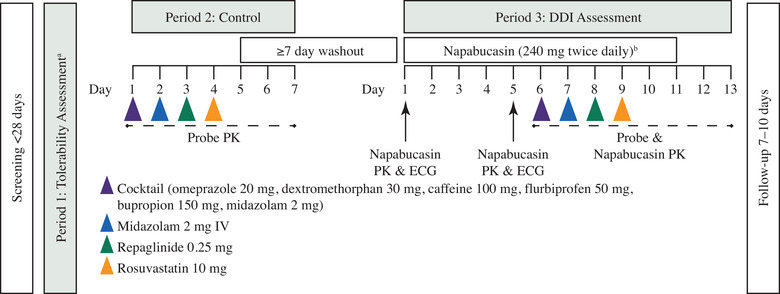
Study design. Dosing schedule of periods 1, 2, and 3. ^a^Healthy volunteers dosed napabucasin in Protocol Amendment 1 received 480 mg twice daily for 2 days (4 doses) in period 1. Healthy volunteers dosed napabucasin in Protocol Amendment 2 received 240 mg twice daily for 2 days (4 doses) in period 1. ^b^Potential DDIs were evaluated in the 17 healthy volunteers dosed with napabucasin 240 mg twice daily under Protocol Amendment 2. Healthy volunteers under Protocol Amendment 1 received a single dose of napabucasin 480 mg and were discontinued, so DDI could not be evaluated. Eleven healthy volunteers who received 480 mg in period 1 and one 480‐mg dose in period 3 before the Protocol Amendment continued receiving 240 mg in period 3 after the Protocol Amendment and were assessed for DDI. DDI, drug‐drug interaction; ECG, electrocardiogram; IV, intravenous; PK, pharmacokinetics.

The study consisted of a screening period, 3 treatment periods, and a safety follow‐up visit occurring 7 to 10 days after the last dose of study drug (Figure 2). Healthy volunteers were screened within 28 days (day –28 to day –1) before admission to the clinical research unit. Baseline assessments were collected on day 0 or on day 1 before dosing.

#### Period 1: Tolerability Assessment

Napabucasin is associated with gastrointestinal (GI) AEs (eg, nausea, vomiting, diarrhea), and some patients who experience severe GI AEs require napabucasin dose hold, modification, or permanent discontinuation.^17^, ^38^ As the ability to assess drug tolerability in healthy volunteers is diminished by these typical napabucasin‐related GI events, only healthy volunteers who could tolerate napabucasin administered on days 1 and 2 of period 1 (no GI Common Terminology Criteria for Adverse Events [CTCAE] grade >1 AE) were permitted to continue on to period 2.

#### Period 2: Control

After a 7‐day washout, healthy volunteers received a single oral dose of a phenotyping cocktail (for DDI probe drugs, see Table 1) on day 1 of period 2. On day 2, IV midazolam 2 mg was administered, and on days 3 to 4, single oral doses of repaglinide 0.25 mg (day 3) and rosuvastatin 10 mg (day 4) were administered, which were followed by another 7‐day washout.

#### Period 3: DDI Assessment

On days 1 to 11 of period 3, napabucasin was administered. On days 6 to 9, healthy volunteers received single doses of the phenotyping cocktail (day 6), IV midazolam (day 7), repaglinide (day 8), and rosuvastatin (day 9). Following period 3, healthy volunteers participated in a safety follow‐up visit 7 to 10 days after administration of the last dose of study drug.

### Safety Analysis

Safety analyses included the incidence of treatment‐emergent AEs (TEAEs), AEs by severity, and serious AEs (SAEs; periods 1‐3). Analyses also included changes from baseline in electrocardiogram parameters, clinical laboratory parameters, urinalysis, vital signs, and changes from predose physical exam findings to study discharge (periods 2 and 3).

### Pharmacokinetic Sampling and Assays

In period 2, blood samples for the PK analysis of each of the probe drugs and their metabolites were obtained before dosing through 24 hours following administration of the phenotyping cocktail, IV midazolam, and repaglinide on days 1, 2, and 3, respectively, and through 72 hours following rosuvastatin administration on day 4.

Blood samples for the determination of napabucasin and M1 metabolite concentrations were collected before dosing, up to 10 hours following the morning doses on day 1 and day 5, and before the morning doses on days 2 to 4 in period 3. Blood samples for the PK analysis of each of the probe drugs were obtained before dosing through 24 hours following administration of the phenotyping cocktail, IV midazolam, and repaglinide on days 6, 7, and 8, respectively, and through 96 hours following rosuvastatin administration on day 9. PK samples collected on days 6 to 9 of period 3 were assayed for napabucasin, probe drugs, probe drug metabolites, and M1. M1 is the major circulating napabucasin metabolite; all other napabucasin metabolites are minor plasma components (≤7%) in healthy adult male human subjects.^39^ Thus, M1 was the metabolite measured in this study.

Napabucasin, M1, and probe drug plasma concentrations were quantified at Charles River Labs in Worcester, Massachusetts, using a validated liquid chromatography with tandem mass spectrometry bioanalytical method (ultra‐performance liquid chromatography with mass spectrometric detection). The lower limit of quantification was 5 ng/mL, and the upper limit of quantification was 500 ng/mL. Detailed descriptions of the PK assays are provided in the Supplemental Methods.

### Pharmacokinetic Analysis

PK parameters for napabucasin, M1, and each of the probe drugs were calculated with Phoenix WinNonlin 6.3 (Certara USA, Inc., Princeton, New Jersey) using actual sampling time and noncompartmental analysis (data permitting). If actual times were missing, nominal times were used. PK parameters included C_max_, time to maximum concentration (t_max_), terminal‐phase half‐life (t_½_), area under the plasma concentration‐time curve (AUC) from time 0 to last measurable plasma concentration (AUC_last_), AUC from time 0 extrapolated to infinity (AUC_inf_), percent AUC extrapolated beyond the last measurable concentration, systemic volume of distribution, and apparent systemic clearance.

Plasma drug concentrations for napabucasin, M1, and probe drugs were summarized using nominal PK sampling times. For the calculation of mean concentrations and generation of mean concentration vs time profiles, all values below the limit of quantification (BLQ) were set to 0.

For the PK analysis, a concentration that was BLQ was assigned a value of 0 if it occurred in a profile before the first measurable concentration. If a BLQ value occurred after a measurable concentration in a profile and was followed by a value above the lower limit of quantification, then the BLQ value was treated as missing data. If a BLQ value occurred at the end of the collection interval (after the last quantifiable concentration), it was treated as missing data. If 2 BLQ values occurred in succession after C_max_, the profile was deemed to have terminated at the first BLQ value, and any subsequent concentrations were omitted from the PK calculations by treating them as missing.

The PK results are reported as both the geometric mean with the associated coefficient of variation (CV%) and the arithmetic mean with the associated standard deviation (SD) or standard error of the mean (SEM) for each observed PK exposure measure with and without napabucasin. The DDI results are reported as geometric mean ratios with associated 90% confidence intervals (CIs). A linear mixed‐effects model was used to perform the DDI analysis comparing intravolunteer systemic exposures of each probe drug in the presence and absence of napabucasin. The analysis was performed on the natural log‐transformed AUC_last_, AUC_inf_, and C_max_ of each probe drug. The model included treatment as a fixed effect and volunteer as a random effect.

Each model included calculation of the least squares mean (LSM), the difference between treatment LSM, and the standard error associated with the difference. The geometric mean ratio of AUC_last_, AUC_inf_, and C_max_, along with the 2‐sided 90%CI, was derived from the LSM difference between each probe drug in the presence and absence of napabucasin in the model. This difference and the associated CI were back‐transformed to provide a ratio of geometric LSM and associated 90%CI. The end points of the bioequivalence interval of 80% to 125% were considered the no‐effect boundaries. If the 90%CI for the measured changes in systemic exposures fell completely within the no‐effect boundaries, it was interpreted that no DDI was present. If any of the 90%CI fell outside of the 80% to 125% range of the established no‐effect boundaries, the interpretation of a potential DDI relied on clinical understanding of the therapeutic range with regard to safety and efficacy.

## Results

### Baseline Demographics and Healthy Volunteer Disposition

A total of 30 healthy volunteers received ≥1 dose of napabucasin 240 or 480 mg between July 6, 2017, and January 23, 2018 (Figure S1). Twenty‐three healthy volunteers in period 1 received napabucasin 480 mg twice daily before the protocol amendment; 22 volunteers received 4 doses over 2 days, and 1 volunteer received 2 doses on day 1 and discontinued due to AEs. Eighteen of the 23 volunteers received a single dose of 480‐mg napabucasin on period 3, day 1, before the study was paused.

A protocol amendment that included formal stopping rules was added, which required termination of dosing in any healthy volunteer experiencing an event that was CTCAE grade ≥2 toxicity and termination of the entire study if ≥2 volunteers experienced any event that was CTCAE grade 3 toxicity or any single volunteer experienced an event that was grade 4 severity. Additionally, per the amendment, the dosing level of napabucasin was lowered from 480 mg twice daily to 240 mg twice daily, as this was the starting dose used in 2 pivotal registration trials (NCT02753127; NCT02993731).

When the study resumed, 11 of the 23 originally enrolled healthy volunteers reconsented and continued the study at the napabucasin 240‐mg dose level in period 3. As a result, these 11 volunteers received napabucasin 480 mg twice daily for 2 days in period 1, 1 dose of napabucasin 480 mg in period 3 before the protocol amendment, and napabucasin 240 mg twice daily for 22 doses over 11 days in period 3 following the protocol amendment. Seven additional healthy volunteers were recruited following the protocol amendment, and received napabucasin 240 mg twice daily in period 1. Of these 7 volunteers, 1 received 1 dose and discontinued prematurely due to AEs; thus, 6 volunteers received napabucasin 240 mg twice daily in period 3.

Of the 30 total healthy volunteers enrolled, 17 were evaluated for DDI potential and constitute the DDI population (Table 2). Of those volunteers in the DDI population, the mean (SD) age was 29 (6.3) years, the majority were men (64.7%) and black or African American (76.5%), and the mean (SD) body mass index was 27.0 (3.29) kg/m^2^. All patients in the DDI population had a normal CYP2C9 genotype, and the majority had normal CYP2D6 and CYP2C19 genotypes (82.4% and 52.9%, respectively).

**Table 2:** Healthy Volunteer Demographics

Characteristic	Safety Population (N = 30)	DDI Population (n = 17)^a^
Age, y, mean (SD)	29 (5.9)	29 (6.3)
Sex, male, n (%)	20 (66.7)	11 (64.7)
Race, n (%)		
Black or African American	16 (53.3)	13 (76.5)
White	14 (46.7)	4 (23.5)
BMI, kg/m^2^, mean (SD)	26.9 (3.9)	27 (3.3)
CYP2D6 genotype, n (%)		
Normal	27 (90)	14 (82.4)
Intermediate	2 (6.7)	2 (11.8)
Ultra‐rapid	1 (3.3)	1 (5.9)
CYP2C9 genotype, n (%)		
Normal	28 (93.3)	17 (100)
Intermediate	2 (6.7)	0
CYP2C19 genotype, n (%)		
Normal	16 (53.3)	9 (52.9)
Intermediate	7 (23.3)	6 (35.3)
Extensive	1 (3.3)	1 (5.9)
Ultra‐rapid	6 (20)	1 (5.9)

BMI, body mass index; CYP, cytochrome P450; DDI, drug‐drug interaction; PK, pharmacokinetics; SD, standard deviation.

^a^ All healthy volunteers with both phenotyping cocktail and napabucasin concentrations.

### Drug‐Drug Interactions

**Figure 3:**
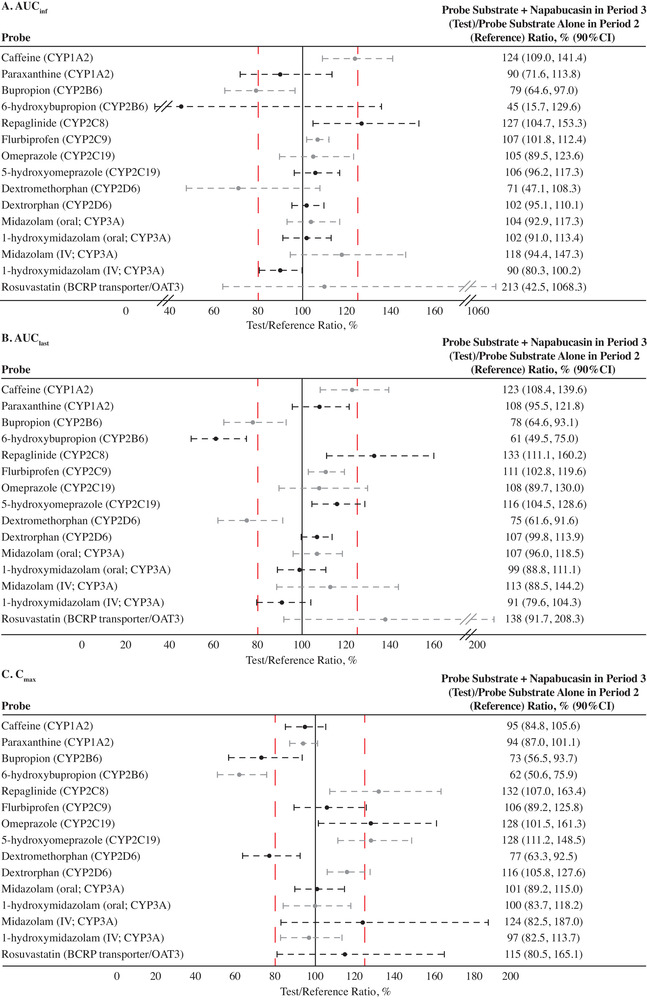
Drug‐drug interactions (DDIs) of napabucasin 240 mg and probe substrates and probe metabolites. Changes in (A) AUC_inf_, (B) AUC_last_, and (C) C_max_. The red dashed lines denote the no‐effect boundaries (80%‐125%). AUC, area under the concentration curve; AUC_inf_, AUC from time 0 extrapolated to infinity; AUC_last_, AUC from time 0 to time of last measurable plasma concentration; BCRP, breast cancer resistance protein; CI, confidence interval; C_max_, maximum observed plasma concentration; CYP, cytochrome P450; IV, intravenous; OAT3, organic anion transporter 3.

**Figure 4:**
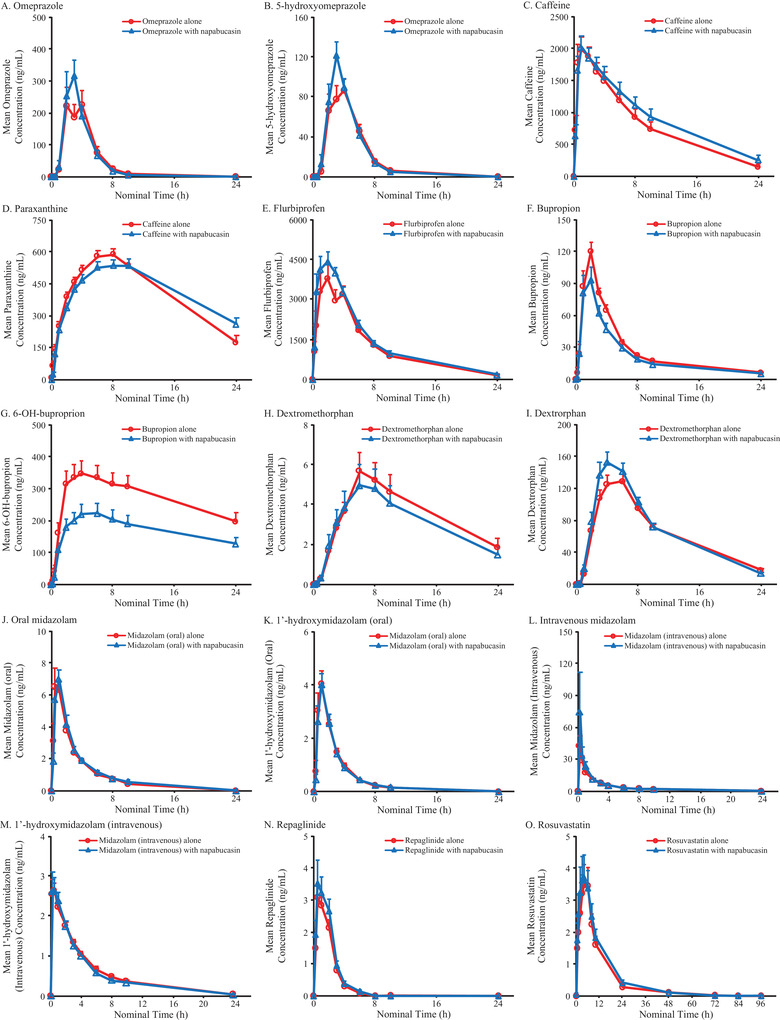
Mean plasma concentrations of probe substrates and probe metabolites over time when administered with (blue line, triangles) or without napabucasin (red line, circles). Mean concentrations of (A) omeprazole and (B) metabolite 5‐hydroxyomeprazole, (C) caffeine and (D) metabolite paraxanthine, (E) flurbiprofen, (F) bupropion and (G) metabolite 6‐OH‐buproprion, (H) dextromethorphan and (I) metabolite dextrorphan, (J) oral midazolam and (K) metabolite 1′‐hydroxymidazolam (oral), (L) intravenous midazolam and (M) metabolite 1′‐hydroxymidazolam (intravenous), (N) repaglinide, and (O) rosuvastatin are shown. Data are plotted as mean ± SEM.

The tested dose level of napabucasin 240 mg twice daily provided sufficient plasma exposure of napabucasin (Table 3) and its primary metabolite napabucasin M1 (Table 4) to assess potential DDI effects. Steady‐state exposure due to administration of napabucasin 240 mg twice daily had no impact on exposure to omeprazole, 5‐hydroxyomeprazole, paraxanthine, flurbiprofen, dextrorphan, oral midazolam, or 1‐hydroxymidazolam (both oral and IV) (Figure 3A‐C, Figure 4).

**Table 3:** Noncompartmental Plasma Pharmacokinetic Parameters of Probes

	Period 2 (Probe Alone)		Period 3 (Probe With Napabucasin)	
	Arithmetic Mean (SD)	Geometric Mean (Geometric %CV)	Arithmetic Mean (SD)	Geometric Mean (Geometric %CV)
Bupropion, oral, CYP2B6				
AUC_last_, ng • h/mL	639 (152)	619 (28.0)	524 (238)	480 (44.0)
AUC_ext_, %	11.4 (6.38)	9.81 (65.9)	11.4 (4.86)	10.5 (42.7)
AUC_inf_, ng • h/mL	727 (186)	701 (30.2)	566 (238)	526 (39.7)
C_max_, ng/mL	134 (38.1)	130 (27.6)	110 (67.7)	94.3 (61.4)
C_last_, ng/mL	6.02 (2.55)	5.45 (51.2)	5.14 (2.62)	4.69 (43.4)
CL/F, L/H	224 (79.9)	214 (30.2)	303 (102)	285 (39.7)
t_½_, h	9.21 (3.24)	8.74 (34.0)	8.77 (1.90)	8.59 (20.7)
Omeprazole, oral, CYP2C19				
AUC_last_, ng • h/Ml	1010 (468)	894 (56.0)	999 (568)	828 (76.6)
AUC_ext_, %	3.16 (5.08)	1.77 (122)	1.85 (0.853)	1.65 (58.4)
AUC_inf_, ng • h/mL	1260 (409)	1190 (37.8)	1220 (495)	1130 (44.1)
C_max_, ng/mL	390 (194)	338 (66.4)	439 (236)	379 (64.1)
C_last_, ng/mL	17.3 (17.1)	13.2 (78.6)	12.9 (7.17)	11.3 (57.9)
CL/F, L/H	17.9 (7.23)	16.8 (37.8)	19.3 (8.38)	17.7 (44.1)
t_½_, h	1.29 (0.448)	1.24 (30.3)	1.10 (0.226)	1.08 (20.5)
Dextromethorphan, oral, CYP2D6				
AUC_last_, ng • h/mL	78.1 (56.6)	60.4 (89.4)	70.0 (64.5)	45.4 (129)
AUC_ext_, %	20.6 (7.33)	19.2 (43.2)	17.3 (7.94)	15.7 (47.7)
AUC_inf_, ng • h/mL	77.1 (46.5)	64.7 (76)	89.9 (102)	48.8 (170)
C_max_, ng/mL	5.85 (3.93)	4.66 (82.9)	5.16 (4.41)	3.57 (115)
C_last_, ng/mL	1.86 (1.84)	1.25 (113)	1.49 (1.70)	0.772 (186)
CL/F, L/H	558 (360)	464 (70.6)	1000 (840)	615 (170)
t_½_, h	9.16 (2.29)	8.90 (26.1)	8.29 (2.35)	8.01 (27.2)
Midazolam, oral, CYP3A				
AUC_last_, ng • h/mL	20.1 (7.62)	19.0 (35.5)	21.3 (7.06)	20.2 (34.3)
AUC_ext_, %	14.3 (6.12)	13.0 (47.8)	13.3 (4.88)	12.5 (37.3)
AUC_inf_, ng • h/mL	23 (8.93)	21.6 (36.9)	24.8 (9.00)	23.4 (36.7)
C_max_, ng/mL	8.01 (4.09)	7.28 (44.4)	7.77 (2.61)	7.37 (34.6)
C_last_, ng/mL	0.65 (0.25)	0.612 (35.5)	0.658 (0.317)	0.607 (40.3)
CL/F, L/H	98 (32.7)	92.6 (36.9)	90.8 (32.4)	85.6 (36.7)
t_½_, h	3.53 (1.46)	3.25 (43.7)	3.5 (1.09)	3.33 (33.5)
Midazolam, IV, CYP3A				
AUC_last_, ng • h/mL	74.7 (19.1)	72.7 (23.3)	94.0 (72.6)	80.9 (53.6)
AUC_ext_, %	11.4 (5.43)	10.2 (53.1)	9.93 (6.41)	7.86 (88.3)
AUC_inf_, ng • h/mL	81.0 (16.1)	79.6 (18.9)	103 (72.2)	90.0 (50.5)
C_max_, ng/mL	42.4 (41.3)	35.2 (54.2)	75.9 (152)	43.7 (91.5)
C_last_, ng/mL	1.49 (0.623)	1.34 (53.7)	1.30 (0.885)	1.03 (82.8)
CL/F, L/H	25.5 (4.61)	25.1 (18.9)	24.3 (9.98)	22.2 (50.5)
t_½_, h	4.57 (1.63)	4.31 (36.3)	5.30 (2.96)	4.77 (46.7)
Vz, L	162 (44.8)	156 (28.9)	189 (139)	153 (80.0)
Caffeine (oral), CYP1A2				
AUC_last_, ng • h/mL	17 300 (10 700)	15 000 (57.4)	21 100 (11 400)	18 400 (59.9)
AUC_ext_, %	8.65 (7.77)	6.13 (105)	9.70 (8.38)	7.45 (83.4)
AUC_inf_, ng • h/mL	20 100 (15 700)	16 800 (62.3)	24 800 (17 900)	20 500 (69.9)
C_max_, ng/mL	2 270 (905)	2 110 (39.6)	2 110 (756)	2 000 (34.6)
C_last_, ng/mL	262 (296)	164 (127)	262 (306)	172 (112)
CL/F, L/H	6.82 (3.45)	5.97 (62.3)	5.91 (4.30)	4.88 (69.9)
t_½_, h	5.19 (2.59)	4.81 (37.9)	6.67 (3.06)	6.15 (42.2)
Flurbiprofen, oral, CYP2C9				
AUC_last_, ng • h/mL	28 700 (8 310)	27 500 (31.1)	31 800 (6 890)	31 100 (22.2)
AUC_ext_, %	3.95 (2.39)	3.46 (53.6)	4.15 (2.36)	3.70 (49.0)
AUC_inf_, ng • h/mL	30 900 (8 360)	29 800 (28.0)	33 300 (7 630)	32 500 (23.5)
C_max_, ng/mL	5 500 (1 840)	5 210 (35.3)	5 770 (1 300)	5 630 (23.4)
C_last_, ng/mL	223 (261)	160 (84.9)	177 (102)	155 (55.2)
CL/F, L/H	1.74 (0.478)	1.68 (28.0)	1.58 (0.367)	1.54 (23.5)
t_½_, h	5.16 (0.822)	5.11 (15.1)	5.43 (0.761)	5.38 (13.0)
Repaglinide, oral, CYP2C8				
AUC_last_, ng • h/mL	4.15 (2.01)	3.72 (51.1)	5.27 (2.54)	4.75 (49.9)
AUC_ext_, %	8.14 (3.67)	7.36 (50.0)	7.51 (4.66)	6.35 (65.1)
AUC_inf_, ng • h/mL	4.45 (1.98)	4.08 (45.1)	6.01 (2.57)	5.53 (45.4)
C_max_, ng/mL	3.38 (1.58)	3.09 (44.5)	4.43 (2.58)	3.85 (58.0)
C_last_, ng/mL	0.282 (0.0539)	0.277 (18.3)	0.332 (0.124)	0.315 (33.5)
CL/F, L/H	66.7 (28.4)	61.3 (45.1)	49.6 (24.1)	45.2 (45.4)
t_½_, h	0.770 (0.183)	0.751 (23.5)	0.795 (0.246)	0.765 (28.2)
Rosuvastatin, oral, BCRP/OAT3‐mediated interactions				
AUC_last_, ng • h/mL	38.4 (25.0)	29.1 (107)	49.3 (35.9)	35.2 (136)
AUC_ext_, %	7.98 (2.71)	7.61 (35.1)	7.03 (5.63)	5.41 (85.5)
AUC_inf_, ng • h/mL	27.8 (19.4)	21.1 (117)	54.1 (36.1)	40.3 (125)
C_max_, ng/mL	3.91 (2.63)	3.03 (93.8)	4.49 (3.08)	3.29 (122)
C_last_, ng/mL	0.202 (0.113)	0.181 (49.3)	0.230 (0.0782)	0.216 (39.9)
CL/F, L/h	693 (738)	475 (117)	518 (1120)	248 (125)
t_½_, h	9.29 (7.36)	7.11 (103)	7.66 (3.53)	6.98 (46.8)

%CV, percent coefficient of variation; AUC, area under the curve; AUC_inf_, AUC from time 0 extrapolated to infinity; AUC_ext_, percent AUC extrapolated beyond the last measurable concentration; AUC_last_, AUC from time 0 to last measurable concentration; BCRP, breast cancer resistance protein; C_last_, last observed quantifiable concentration; CL/F, apparent systemic clearance; C_max_, maximum concentration; CYP, cytochrome P450; DDI, drug‐drug interaction; IV, intravenous; LS, least squares; OAT3, organic anion transporter 3; SD, standard deviation; t_½_, half‐life; Vz, systemic volume of distribution following IV administration.

**Table 4:** Plasma Noncompartmental PK Parameters of Probe Metabolites in Periods 2 and 3

	Period 2 (Probe Alone)		Period 3 (Probe With Napabucasin)	
	Arithmetic Mean (SD)	Geometric Mean (Geometric %CV)	Arithmetic Mean (SD)	Geometric Mean (Geometric %CV)
6‐hydroxybupropion, oral, CYP2B6				
AUC_last_, ng • h/mL	6 310 (3 110)	5 630 (53.3)	4 020 (2 310)	3 430 (65.0)
AUC_ext_, %	33.2 (3.39)	33.1 (10.5)	36.9 (0.373)	36.9 (1.01)
AUC_inf_, ng • h/mL	6 210 (3 730)	5 570 (60.1)	3 340 (2 030)	2 950 (66.8)
C_max_, ng/mL	358 (165)	323 (50.6)	232 (125)	200 (63.4)
C_last_, ng/mL	197 (112)	169 (64.1)	128 (76.4)	108 (69.1)
t_½_, h	13.9 (1.13)	13.9 (8.32)	16.4 (1.28)	16.3 (7.66)
5‐hydroxyomeprazole, oral, CYP2C19				
AUC_last_, ng • h/mL	388 (112)	373 (29.6)	444 (110)	433 (23.5)
AUC_ext_, %	2.25 (0.974)	2.07 (44.1)	2.44 (1.33)	2.20 (46.0)
AUC_inf_, ng • h/mL	431 (110)	420 (23.1)	457 (114)	445 (23.9)
C_max_, ng/mL	124 (49.7)	116 (41.3)	153 (38.8)	148 (26.5)
C_last_, ng/mL	6.88 (4.58)	5.79 (63.0)	5.81 (2.53)	5.34 (43.3)
t_½_, h	1.36 (0.242)	1.34 (17.8)	1.35 (0.190)	1.33 (14.9)
Dextrorphan, oral, CYP2D6				
AUC_last_, ng • h/mL	1 400 (357)	1 350 (29.7)	1 490 (346)	1 440 (26.6)
AUC_ext_, %	11.9 (7.23)	10.3 (56.4)	8.34 (6.42)	6.36 (89.3)
AUC_inf_, ng • h/mL	1 590 (382)	1 540 (28.2)	1 610 (331)	1 580 (22.2)
C_max_, ng/mL	137 (40.8)	130 (36.5)	162 (58.2)	151 (43.0)
C_last_, ng/mL	17.6 (6.48)	16.4 (42.2)	13.5 (5.97)	12.3 (47.1)
t_½_, h	6.97 (2.06)	6.73 (27.0)	5.88 (1.89)	5.63 (30.3)
1‐hydroxymidazolam, oral, CYP3A				
AUC_last_, ng • h/mL	10.9 (3.86)	10.2 (44.8)	10.7 (3.67)	10.1 (34.8)
AUC_ext_, %	5.95 (3.31)	5.12 (62.5)	6.37 (2.77)	5.78 (49.8)
AUC_inf_, ng • h/mL	11.6 (3.97)	10.8 (43.1)	11.6 (3.95)	11.0 (35.3)
C_max_, ng/mL	4.55 (2.12)	4.08 (52.9)	4.45 (2.15)	4.06 (44.5)
C_last_, ng/mL	0.164 (0.0544)	0.157 (29.7)	0.187 (0.0636)	0.177 (34.4)
t_½_, h	2.62 (1.09)	2.44 (39.8)	2.59 (0.812)	2.47 (33.9)
1‐hydroxymidazolam, IV, CYP3A				
AUC_last_, ng • h/mL	11.4 (3.04)	11.0 (31.2)	10.7 (3.32)	10.2 (33.6)
AUC_ext_, %	15.1 (6.30)	14.0 (41.4)	14.8 (7.21)	13.0 (62.4)
AUC_inf_, ng • h/mL	13.5 (3.41)	13.0 (29.9)	12.1 (3.18)	11.7 (28.8)
C_max_, ng/mL	2.94 (1.17)	2.72 (43.8)	3.05 (1.72)	2.75 (46.0)
C_last_, ng/mL	0.286 (0.139)	0.253 (57.3)	0.287 (0.114)	0.265 (44.9)
t_½_, h	5.67 (2.88)	5.06 (52.3)	4.41 (2.17)	4.02 (44.4)
Paraxanthine, oral, CYP1A2				
AUC_last_, ng • h/mL	8 930 (2 500)	8 560 (31.9)	9 550 (2 130)	9 240 (29.8)
AUC_ext_, %	12.8 (8.79)	10.3 (81.7)	18.9 (8.15)	17.6 (47.2)
AUC_inf_, ng • h/mL	9 680 (3 190)	9 230 (33.6)	7 910 (2 430)	7 610 (33.5)
C_max_, ng/mL	610 (97.2)	603 (15.7)	572 (89.6)	566 (14.9)
C_last_, ng/mL	204 (148)	157 (90.7)	271 (111)	247 (49.7)
t_½_, h	7.03 (2.47)	6.68 (34.7)	7.40 (2.13)	7.19 (27.9)

%CV, percent coefficient of variation; AUC, area under the curve; AUC_inf_, AUC from time 0 to infinity; AUC_ext_, percent AUC extrapolated beyond the last measurable concentration; AUC_last_, AUC from time 0 to last measurable concentration; C_last_, last observed quantifiable concentration; CL/F, apparent systemic clearance; C_max_, maximum concentration; CYP, cytochrome P450; DDI, drug‐drug interaction; IV, intravenous; LS, least squares; OAT3, organic anion transporter 3; SD, standard deviation; t_½_, half‐life.

Coadministration of napabucasin increased the AUC_inf_ (geometric mean ratio as percentage [90%CI]) of caffeine (124% [109.0%‐141.4%]), IV midazolam (118% [94.4%‐147.3%]), repaglinide (127% [104.7%‐153.3%]), and rosuvastatin (213% [42.5%‐1 068.3%]) and decreased the AUC_inf_ of dextromethorphan (71% [47.1%‐108.3%]), bupropion (79% [64.6%‐97.0%]), and 6‐hydroxybupropion (45% [15.7%‐129.6%]) (Figure 3A). Similar results were found for AUC_last_ (Figure 3B; and presented as arithmetic means [SEM] in Figure 4).

Coadministration of napabucasin increased the C_max_ (geometric mean ratio as percentage [90%CI]) of caffeine (95% [84.8%‐105.6%]), IV midazolam (124% [82.5%‐187.0%]), repaglinide (132% [107.0%‐163.4%]), and rosuvastatin (115% [80.5%‐165.1%]) and decreased the C_max_ of dextromethorphan (77% [63.3%‐92.5%]), bupropion (73% [56.5%‐93.7%]), and hydroxybupropion (62% [50.6%‐75.9%]) (Figure 3C; and presented as arithmetic means [SEM] in Figure 4).

Generally, napabucasin did not induce drug clearance to a clinically meaningful degree for all measured components, with the possible exception of hydroxybupropion, for which post‐napabucasin AUC exposures were 45% of control (representing a 55% reduction). Per the US Food and Drug Administration, reductions between 50% and 80% are considered moderate reduction effects.^40^ This may be a result of decreased formation of hydroxybupropion from bupropion, rather than an induction of elimination.

### Pharmacokinetics—Period 3

Among healthy volunteers who received a single dose of napabucasin 240 mg, the geometric mean C_max_ (CV%) was 375 (26.6) ng/mL, and the geometric mean AUC_last_ (CV%) was 1660 (28.8) ng • h/mL. PK parameters following repeated napabucasin 240 mg twice‐daily administration were comparable across all days sampled (days 5‐9) (Table S1), with geometric mean C_max_ ranging from 287 to 652 ng/mL, geometric mean AUC_last_ ranging from 1570 to 4160 ng • h/mL, and median t_max_ ranging from 2.98 to 5.00 hours. Geometric mean t_½_ ranged from 1.64 to 2.66 hours (Table S1).

Similar results were observed for key napabucasin M1 PK parameters (Table S1), with geometric mean C_max_ ranging from 226 to 544 ng/mL, geometric mean AUC_last_ ranging from 1 290 to 3 690 ng • h/mL, and the median t_max_ value ranging from 2.46 to 6.00 hours. Geometric mean t_½_ ranged from 1.54 to 2.68 hours (Table S1).

### Safety

TEAEs were experienced by most healthy volunteers while receiving repeated napabucasin doses in period 1 (240‐mg or 480‐mg doses: 70.0% [21/30 volunteers]) and period 3 (240‐mg dose: 70.6% [12/17 volunteers]), and most were grade 1 (experienced by 63.3% [19/30] of volunteers in period 1 and 37.5% [9/24] in period 3) or grade 2 in severity (experienced by 6.7% [2/30] of volunteers in period 1 and 29.2% [7/24] in period 3) (Table 5). Two volunteers experienced grade 2 AEs after the protocol amendment; however, treatment was not discontinued because the events were originally reported as grade 1. Study monitoring queried the grade of these 2 AEs because the source data described the AEs as “moderate.” Based on these queries, the site changed the grade to grade 2. This occurred after the volunteers had already completed the study. All other grade 2 events (n = 7 patients) occurred before the amendment and therefore before the requirement to stop treatment. Only 3 of 26 healthy volunteers had a TEAE during period 2, when napabucasin was not administered. Overall, 19 volunteers in period 1 and 9 in period 3 experienced mild (grade 1) TEAEs. One grade 3 TEAE (severe; low neutrophil count) was observed during period 3 in a healthy volunteer who was reenrolled after the study was paused. The volunteer's neutrophil count was toward the low range of normal (1.4 × 10^9^ cells/L) at rescreening, when the study resumed, and decreased to 0.9 × 10^9^ cells/L at follow‐up after completion of period 3.

**Table 5:** Summary of TEAEs Regardless of Relationship to Study Drug in Periods 1 and 3

	Period 1			Period 3		
Summary of TEAEs, Healthy Volunteers, n (%)	All (N = 30)	Napabucasin 240 mg Twice Daily (n = 7)	Napabucasin 480 mg Twice Daily (n = 23)	All (N = 24)^a^	Napabucasin 240 mg Twice Daily (n = 17)	Napabucasin 480 mg (n = 18)^b^
≥1 TEAE	21 (70.0)	5 (71.4)	16 (69.6)	17 (70.8)	12 (70.6)	11 (61.1)
Severity						
Grade 1	19 (63.3)	4 (57.1)	15 (65.2)	9 (37.5)	9 (52.9)	4 (22.2)
Grade 2	2 (6.7)	1 (14.3)	1 (4.3)	7 (29.2)	2 (11.8)	7 (38.9)
Grade 3	0	0	0	1 (4.2)^c^	1 (5.9)^c^	0
Healthy volunteers with TEAEs, n (%)						
Diarrhea	14 (46.7)^d^	4 (57.1)^e^	10 (43.5)^f^	15 (62.5)	9 (52.9)^g^	10 (55.6)^h^
Chromaturia	9 (30.0)	1 (14.3)	8 (34.8)	1 (4.2)	1 (5.9)	0
Abdominal pain	8 (26.7)	0	8 (34.8)	6 (25.0)	0	6 (33.3)
Vomiting	4 (13.3)	1 (14.3)	3 (13)	2 (8.3)	2 (11.8)	1 (5.6)
Nausea	2 (6.7)	0	2 (8.7)	3 (12.5)	3 (17.6)	0
Abdominal pain, upper	1 (3.3)	1 (14.3)	0	0	0	0
Diarrhea, hemorrhagic	0	0	0	1 (4.2)	1 (5.9)	0
Headache	0	0	0	3 (12.5)	2 (11.8)	1 (5.6)
Pollakiuria	0	0	0	1 (4.2)	1 (5.9)	0

TEAE, treatment emergent adverse event.

^a^ All healthy volunteers enrolled in period 3 include the 7 enrolled after the protocol amendment who always received the 240‐mg dose and the 18 enrolled before the protocol amendment who received one 480‐mg dose in period 3.

^b^ Eighteen healthy volunteers who were administered napabucasin in the protocol amendment received a single 480‐mg dose on day 1, period 3, and resumed dosing in period 3 at napabucasin 240 mg twice daily. Exposure of healthy volunteers to napabucasin 480 mg in period 3 was for one dose only.

^c^ Low neutrophil count (severe) was observed in a healthy volunteer who was reenrolled after the study was paused.

^d^ Thirteen grade 1 and 1 grade 2.

^e^ All grade 1.

^f^ Nine grade 1 and 1 grade 2.

^g^ Eight grade 1 and 1 grade 2.

^h^ Three grade 1 and 7 grade 2.

The most frequent TEAEs reported in >1 healthy volunteer who received napabucasin 240 mg twice daily were diarrhea (57.1% [4/7]) in period 1 and diarrhea (52.9% [9/17]), nausea (17.6% [3/17]), headache (11.8% [2/17]), and vomiting (11.8% [2/17]) in period 3 (Table 5). All events resolved, except for the event of low neutrophil count (severe) in 1 volunteer for which the outcome was unknown (ie, no additional hematologic laboratory values were obtained for this volunteer). Two healthy volunteers experienced TEAEs that led to study withdrawal: 1 occurred during the tolerability assessment period (period 1) in a volunteer who had received 2 doses of napabucasin 480 mg and experienced nausea and diarrhea (both grade 2; both resolved 13 days after study drug discontinuation); the other occurred in a volunteer who received 1 dose of napabucasin 240 mg in period 1 and experienced vomiting (grade 2; resolved 15 minutes after it began) and stomach pain (grade 2; resolved 2 hours after it began). No deaths or SAEs were reported, and no clinically meaningful changes from baseline were observed in vital signs or electrocardiogram results.

## Discussion

DDIs are common in patients undergoing cancer treatment.^21^ Risk factors for potential DDIs include the number and type of medications used and age‐related changes in metabolism.^41^, ^42^, ^43^, ^44^ Most drugs, including anticancer drugs, are primarily metabolized by CYPs, and the majority of DDIs are due to inhibition or induction of different CYPs.^45^

In vitro, napabucasin is an inhibitor of the CYP2C9, CYP2C19, CYP3A, CYP1A2, CYP2D6, CYP2C8, and CYP2B6 isozymes, as well as the BCRP/OAT3 (Sumitomo Dainippon Pharma Oncology, Inc., data on file). This was the first study to quantitatively assess the in vivo DDI potential of napabucasin with respect to these major human drug CYP enzymes and the BCRP/OAT3. This study employed a phenotyping cocktail, which permitted the simultaneous but independent evaluation of multiple probe drugs in the same healthy volunteer; this design reduced the study duration, number of volunteers needed, and intervolunteer variability.^46^, ^47^, ^48^

The results from this study suggest minimal in vivo DDI potential of napabucasin or its metabolite with respect to the 7 major human CYP enzymes and the BCRP/OAT3. DDI results were interpreted as clinically meaningful if the magnitude of the exposure change was >2‐fold, a common cutoff used to define a clinically significant effect.^40^ The exposure of bupropion (substrate of CYP2B6) and its metabolite hydroxybupriopion decreased when coadministered with napabucasin; the magnitude of decrease was <2‐fold for bupropion and slightly >2‐fold for hydroxybupriopion. The exposure of rosuvastatin (BCRP/OAT3) increased following coadministration of napabucasin, but these changes are not expected to be clinically meaningful. Collectively, except for the sensitive substrate of CYP2B6 (bupropion), whereby the metabolite hydroxybupriopion exposure changed by 55%, the changes observed with respect to the other 6 major human CYP enzymes and the BCRP/OAT3 are not expected to be clinically meaningful because their DDI did not result in a change that exceeded a factor of 2.

As patients with cancer often receive combination treatment, it is important to diminish DDI effects and maintain the optimal exposure of each cancer agent. In this study, the PK of napabucasin and M1 were comparable across all days sampled in period 3, when both napabucasin 240 mg twice daily and the probe drugs were administered. A phase 1 study of 6 Japanese patients with advanced/recurrent gastric cancer (JapicCTI‐142420) found that the PK and safety profiles of napabucasin were not affected by paclitaxel, a CYP2C8 and CYP3A4 substrate.^18^, ^49^, ^50^ A phase 1b study of napabucasin combined with gemcitabine and the CYP2C8 and CYP3A4 substrate nab‐paclitaxel in patients with metastatic pancreatic adenocarcinoma (NCT02231723; N = 59) also showed no notable PK interactions or dose‐limiting toxicities.^8^, ^49^, ^50^

There were no SAEs, deaths, or fatal AEs reported in this study. The majority of reported TEAEs involved the GI system and were mild to moderate in severity, which is similar to the safety profile reported in previous studies of napabucasin.^8^, ^18^, ^51^ These results suggest that napabucasin is generally tolerable in healthy volunteers when administered at a dose of 240 mg twice daily.

### Limitations

Bupropion (CYP2B6 probe) inhibits CYP2D6 activity (as measured by dextromethorphan in this study) and therefore could confound the DDI potential of napabucasin. However, previous phenotyping cocktail studies showed a low risk for clinically relevant DDI following single‐dose administration.^30^ Furthermore, we observed a decrease rather than an increase in exposure to dextromethorphan and bupropion.

## Conclusions

Generally, per the data from this study in healthy volunteers, napabucasin shows possible moderate inhibition of CYP2B6 vis‐à‐vis metabolite exposure, but it is not expected to induce or inhibit CYP1A2, 2C8, 2C9, 2C19, 2D6, 3A, or the BCRP/OAT3 to a clinically meaningful degree. Coadministration of napabucasin with CYP and transporter substrates was tolerable and associated with a low incidence of grade 3 TEAEs.

## Conflicts of Interest

X.D., M.D.K., and M.H. are employees of Sumitomo Dainippon Pharma Oncology, Inc. C.F.M. and S.J.B. were paid consultants of Boston Biomedical, Inc (now Sumitomo Dainippon Pharma Oncology, Inc.). M.L.H. and M.T.G. previously served as paid consultants to Boston Biomedical, Inc (currently Sumitomo Dainippon Pharma Oncology, Inc.).

## Funding

This study was funded by Sumitomo Dainippon Pharma Oncology, Inc.

## Supporting information

Supplementary information
