# Supplementary material for: Napabucasin Drug‐Drug Interaction Potential, Safety, Tolerability, and Pharmacokinetics Following Oral Dosing in Healthy Adult Volunteers
Source: Clin Pharmacol Drug Dev. 2021 Jun 9;10(8):824–39. doi: 10.1002/cpdd.961 (PMC8453567; doi:10.1002/cpdd.961)

**Supplementary Methods**

**Quantification of napabucasin, M1, probe drugs, and probe metabolites in plasma**

Plasma concentrations of napabucasin, M1, and probe drugs were quantified using Good Laboratory Practice (GLP) at Charles River Laboratories in Worcester, Massachusetts. Napabucasin and M1 were extracted using a liquid-liquid extraction procedure; all probe drugs and probe metabolites were extracted using a protein precipitation extraction procedure. Plasma concentrations were quantified using liquid chromatography with tandem mass spectrometry (SCIEX API-5500, Framingham, MA, USA). Internal standards were added to each sample during extraction. Samples were separated on a 2.1 × 50 mm ACE 3 C18 100A, 3-µm column (Avantor, Radnor, PA, USA). Specific details for each assay are listed in Table S2 and S3. Accuracy and precision values are shown in Table S4 and were within the acceptance criteria, with the exception of 1’-hydroxymidazolam, where 9/12 (75%) batches met acceptance criteria.

**Supplementary Figure Legends**

**Figure S1.** CONSORT diagram (healthy volunteer disposition). ^a^Healthy volunteers dosed with napabucasin in Protocol Amendment 1 received a single 480-mg dose on Day 1, Period 3. ^b^Healthy volunteer requested to discontinue after completing all PK and DDI assessments, and thus is still included in the DDI population. AE, adverse event; BID, twice daily; CONSORT, Consolidated Standards of Reporting Trials; DDI, drug-drug interaction; HV, healthy volunteer; PK, pharmacokinetics.

**Table S1.** Summary of Key Napabucasin and M1 Noncompartmental PK Parameters Following Repeated Administration of Napabucasin 240 mg in Period 3

| Parameter | Day 5 | |  | Day 6 | |  | Day 7 | |  | Day 8 | |  | Day 9 | |
| --- | --- | --- | --- | --- | --- | --- | --- | --- | --- | --- | --- | --- | --- | --- |
|  | Napabucasin  (n = 17) | M1  (n = 17) |  | Napabucasin  (n = 17) | M1  (n = 17) |  | Napabucasin  (n = 17) | M1  (n = 17) |  | Napabucasin  (n = 16) | M1  (n = 16) |  | Napabucasin  (n = 16) | M1  (n = 16) |
| C_max_, ng/mL |  |  |  |  |  |  |  |  |  |  |  |  |  |  |
| Arithmetic mean (SD) | 468 (122) | 436 (138) |  | 686 (227) | 576 (192) |  | 475 (222) | 453 (182) |  | 355 (138) | 326 (149) |  | 366 (160) | 279 (152) |
| Geometric mean (geometric CV%) | 452 (29.0) | 414 (34.6) |  | 652 (34.3) | 544 (37.1) |  | 414 (69.0) | 390 (80.1) |  | 324 (50.4) | 292 (53.1) |  | 287 (136) | 226 (96.0) |
| 95% CI | 390–523 | 348–492 |  | 549–773 | 452–654 |  | 300–570 | 272–561 |  | 252–418 | 224–381 |  | 166–496 | 147–348 |
| t_max_, h |  |  |  |  |  |  |  |  |  |  |  |  |  |  |
| Median | 5.00 | 3.98 |  | 3.98 | 5.98 |  | 3.98 | 3.97 |  | 4.97 | 6.00 |  | 2.98 | 2.46 |
| Range | 1.0–8.0 | 1.0–12.0 |  | 1.0–8.0 | 2.0–8.0 |  | 0–6.4 | 0.2–12.0 |  | 0–12.0 | 0.3–12.0 |  | 0–8.1 | 0–10.0 |
| AUC_last_, h × ng/mL |  |  |  |  |  |  |  |  |  |  |  |  |  |  |
| Arithmetic mean (SD) | 2760 (1170) | 2780 (1070) |  | 4410 (1610) | 3970 (1480) |  | 2850 (1450) | 2800 (1270) |  | 2200 (1330) | 1940 (1120) |  | 2240 (1210) | 1780 (1020) |
| Geometric mean (geometric CV%) | 2550 (43.9) | 2580 (43.0) |  | 4160 (36.7) | 3690 (41.9) |  | 2390 (88.2) | 2300 (104) |  | 1820 (74.4) | 1630 (70.7) |  | 1570 (212) | 1290 (160) |
| 95% CI | 2050–3160 | 2090–3190 |  | 3470–5000 | 3000–4540 |  | 1620–3530 | 1480–3580 |  | 1280–2590 | 1160–2290 |  | 784–3150 | 706–2350 |
| t_½_, h^a^ |  |  |  |  |  |  |  |  |  |  |  |  |  |  |
| Arithmetic mean (SD) | 2.08 (NC) | 2.30 (NC) |  | 2.78 (0.889) | 2.80 (0.925) |  | 2.09 (1.32) | 1.86 (1.19) |  | 1.71 (0.510) | 1.84 (0.546) |  | 1.98 (0.998) | 2.46 (1.60) |
| Geometric mean (geometric CV%) | NC (NC) | NC (NC) |  | 2.66 (34.1) | 2.68 (35.7) |  | 1.74 (75.9) | 1.54 (75.7) |  | 1.64 (33.1) | 1.78 (30.8) |  | 1.82 (47.0) | 2.16 (59.1) |
| 95% CI | NC | NC |  | 1.8–4.0 | 1.7–4.1 |  | 0.9–3.5 | 0.8–3.1 |  | 1.0–2.8 | 0.8–3.8 |  | 1.0–3.2 | 0.9–5.2 |

AUC_last_, area under the curve from time 0 to last measurable concentration; CI, confidence interval; C_max_, maximum concentration; CV%, coefficient of variation; NC, not calculated; SD, standard deviation; t_½_, half-life; t_max_, time to peak plasma concentration.

^a^n=2–6.

**Table S2.** Analytical Assay Details for Quantification of Napabucasin, M1, Probe Drugs, and Probe Metabolites in Plasma

| Analyte | Internal standard | | | Range of quantification (ng/ml) | m/z monitored (Da) |
| --- | --- | --- | --- | --- | --- |
|  | Product (Manufacturer) | Volume (μl)/sample | Concentration (ng/ml) |  |  |
| Napabucasin | Napabucasin (Cambridge Major Laboratories, Inc., Germantown, WI, USA) | 25 | 300 | 5‒500 | 241.0/199.0 |
| M1 | BBI608-d4 (Cambridge Major Laboratories, Inc.) | 25 | 300 | 5‒500 | 243.1/201.0 |
| Omeprazole | esomeprazole-d3 (Toronto Research Chemicals, Toronto, ON, Canada) | 200 | 20 | 5‒2,000 | 346.1/198.1 |
| 5-hydroxyomeprazole | 5-hydroxyomeprazole-d3 (Toronto Research Chemicals) | 200 | 20 | 2.5‒1,000 | 362.1/214.1 |
| Caffeine | caffeine-13C3 (Cerilliant, Round Rock, TX, USA) | 200 | 50 | 25‒10,000 | 195.1/138.1 |
| Paraxanthine | paraxanthine-d3 (Toronto Research Chemicals) | 200 | 50 | 25‒10,000 | 181.1/124.0 |
| Flurbiprofen | flurbiprofen-d3 (Toronto Research Chemicals) | 200 | 100 | 50‒25,000 | 242.9/199.0 |
| Bupropion | bupropion-D9 (Sigma-Aldrich, St. Louis, MO, USA) | 200 | 10 | 1‒400 | 240.1/131.1 |
| 6-OH-buproprion | Hydroxybupropion-D6 (Sigma-Aldrich) | 200 | 10 | 1‒400 | 256.1/139.1 |
| Dextromethorphan | dextromethorphan-d3 (Cerilliant) | 250 | 0.2 | 0.025‒10 | 272.2/215.1 |
| Dextrorphan | dextrorphan-d3 (Cerilliant) | 250 | 4 | 2.5‒1,000 | 259.2/202.1 |
| Midazolam | midazolam-d4 (Cerilliant) | 250 | 4 | 0.4‒200 | 326.1/223.1 |
| 1’-hydroxymidazolam | α-hydroxymidazolam-d4 (Cerilliant) | 250 | 4 | 0.1‒50 | 342.1/203.1 |
| Repaglinide | repaglinide-ethyl-d5 (Toronto Research Chemicals) | 300 | 1 | 0.2‒200 | 453.4/230.2 |
| Rosuvastatin | rosuvastatin-d6 (Toronto Research Chemicals) | 300 | 5 | 0.1‒100 | 482.2/258.3 |

**Table S3.** LC Conditions for Quantification of Napabucasin, M1, Probe Drugs, and Probe Metabolites in Plasma

| Analyte | LC Conditions | | | | |
| --- | --- | --- | --- | --- | --- |
|  | Mobile phase A (v:v) | Mobile phase B (v:v) | Flow rate (ml/min) | Gradient | Data window |
| Napabucasin | 0.1:100 formic acid in water | 0.1:100 formic acid in acetonitrile | 0.5 | 30% B at 0.02 min (step)  50% B at 2.00 min (ramp)  95% B at 3.95 min (step)  30% B at 5.45 min (step) | Start: 0.70 min  Length: 2.00 min |
| M1 |  |  |  |  |  |
| Omeprazole | 0.1:100 formic acid in water | 0.1:100 formic acid in acetonitrile | 0.5  0.7  0.7  0.7  0.5 | 88% A at 1.75 min (step)  25% A at 3.42 min (ramp)  25% A at 3.67 min (step)  5% A at 4.17 min (step)  88% A at 5.17 min (step) | Start: 1.0 min  Length: 2.9 min |
| 5-hydroxyomeprazole |  |  |  |  |  |
| Caffeine |  |  |  |  |  |
| Paraxanthine |  |  |  |  |  |
| Flurbiprofen | 0.1:100 formic acid in water | 0.1:100 formic acid in acetonitrile | 0.6 | 50% B at 0.25 min (step)  85% B at 1.50 min (ramp)  95% B at 2.17 min (step)  50% B at 3.25 min (step) | Start: 0.30 min  Length: 1.40 min |
| Bupropion | 0.1:95:5 formic acid in water and acetonitrile | 0.1:50:50 formic acid in acetonitrile and methanol | 0.7 | 15% B at 0.10 min (step)  40% B at 1.10 min (ramp)  40% B at 1.60 min (step)  95% B at 2.40 min (step)  15% B at 3.20 min (step) | Start: 0.60 min  Length: 1.50 min |
| 6-OH-buproprion |  |  |  |  |  |
| Dextromethorphan | 0.1:95:5 formic acid in water and acetonitrile | 0.1:50:50 formic acid in acetonitrile and methanol | 0.7 | 80% A at 0.02 min (step)  45% A at 1.52 min (ramp)  45% A at 1.77 min (step)  5% A at 2.77 min (step)  80% A at 3.77 min (step) | Start: 0.40 min  Length: 1.40 min |
| Dextrorphan |  |  |  |  |  |
| Midazolam | 1 mM ammonium acetate in 80:20 water and acetonitrile | 0.1:50:50 formic acid in acetonitrile and methanol | 0.6 | 0% B at 1.25 min (step)  20% B at 1.27 min (step)  27.5% B at 3.27 min (ramp)  95% B at 4.35 min (step)  0% B at 5.60 min (step) | Start: 0.8 min Length: 3.0 min |
| 1’-hydroxymidazolam |  |  |  |  |  |
| Repaglinide | 0.1:100 formic acid in water | 0.1:100 formic acid in acetonitrile | 0.7 | 40% B at 0.25 min (step)  80% B at 1.50 min (ramp)  95% B at 2.00 min (step)  40% B at 3.00 min (step) | Start: 0.35 min  Length: 1.00 min |
| Rosuvastatin | 0.1:100 formic acid in water | 0.1:100 formic acid in acetonitrile | 0.6 | 30% B at 0.25 min (step)  65% B at 2.25 min (ramp)  95% B at 3.25 min (step)  30% B at 4.25 min (step) | Start: 0.75 min Length: 2.00 min |

**Table S4.** Accuracy and precision of napabucasin, M1, Probe Drugs, and Probe Metabolite measurements

|  | Intra-run | |  | Inter-run | |
| --- | --- | --- | --- | --- | --- |
|  | Bias | CV |  | Bias | CV |
| Napabucasin | −4.2–5.8% | 2.4–14.1% |  | −1.3–2.8% | 3.0–9.5% |
| M1 | −8.0–4.4% | 2.0–10.8% |  | −6.2–−0.4% | 5.2–8.6% |
| Omeprazole | -5.6–14.8 | 1.3–8.4 |  | -0.6–10.2 | 4.4–5.8 |
| 5-hydroxyomeprazole | -11.6–7.5 | 1.0–15.8 |  | -4.8–1.9 | 5.1–13.4 |
| Caffeine | -10.4–18.8 | 1.3–10.3 |  | -4.6–9.2 | 5.0–10.3 |
| Paraxanthine | -4.8–15.2 | 1.7–11.6 |  | -0.3–4.8 | 4.4–11.6 |
| Flurbiprofen | -13.0–5.0 | 2.1–8.9 |  | -9.0–3.5 | 4.8–7.5 |
| Bupropion | -5.3– -0.6 | 1.0–4.2 |  | -4.3– -2.7 | 1.7–4.0 |
| 6-OH-buproprion | -3.0–12.0 | 1.9–7.7 |  | -2.3–7.0 | 2.7–6.8 |
| Dextromethorphan | -8.4–4.0 | 2.1–10.6 |  | -4.1– -0.5 | 2.9–8.3 |
| Dextrorphan | -7.2–4.8 | 1.7–12.9 |  | -3.2– -0.3 | 2.4–8.0 |
| Midazolam | -14.4–8.3 | 2.4–16.5 |  | -3.1–1.7 | 8.6–12.4 |
| 1’-hydroxymidazolam | -16.7–19.0 | 1.6–12.1 |  | 2.0–5.0 | 6.3–17.5 |
| Repaglinide | 0.8–15.5 | 1.4–12.2 |  | 5.7–11.0 | 3.5–8.8 |
| Rosuvastatin | -2.4–10.7 | 0.8–12.3 |  | 2.1–5.7 | 3.5–5.9 |

CV, coefficient of variation.

**Figure S1.**


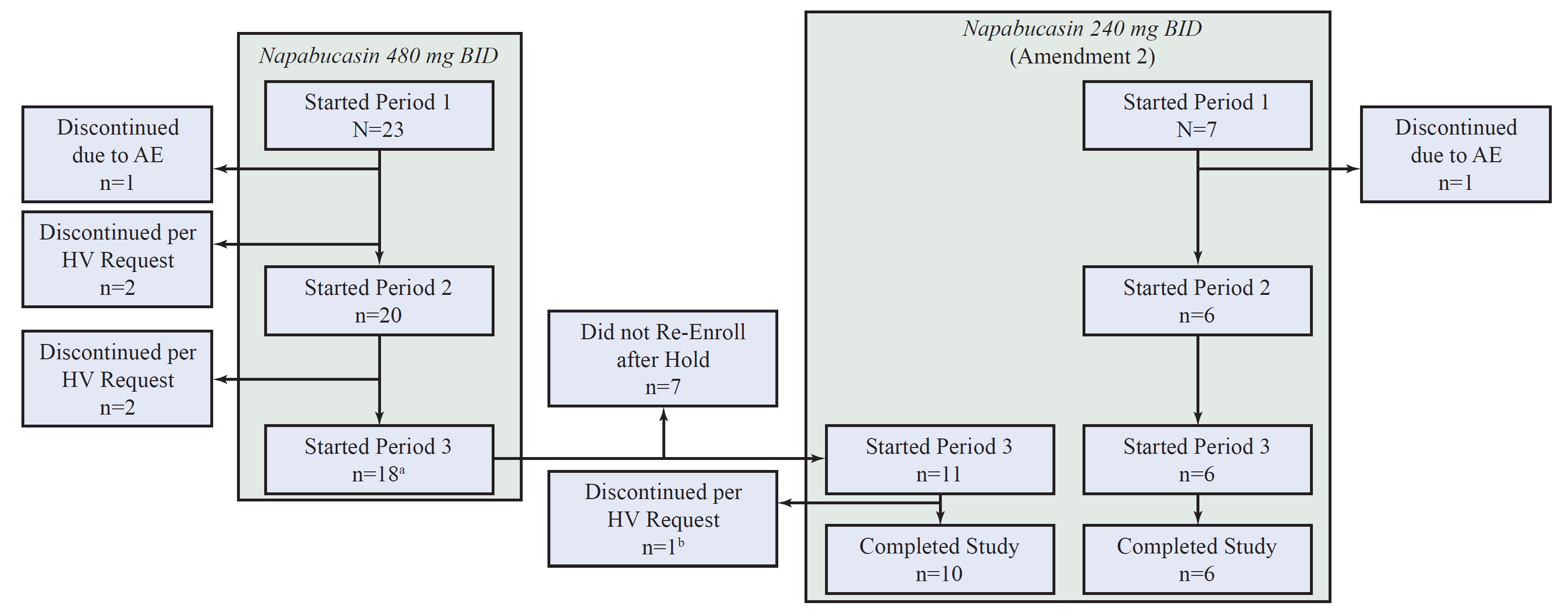

Supplement: Supplementary file 1 — Supplementary information [file CPDD-10-824-s001.docx]
